# The impact of chronic co-exposure to different heavy metals on small fibers of peripheral nerves. A study of metal industry workers

**DOI:** 10.1186/s12995-021-00302-6

**Published:** 2021-04-15

**Authors:** Magdalena Koszewicz, Katarzyna Markowska, Marta Waliszewska-Prosol, Rafał Poreba, Paweł Gac, Anna Szymanska-Chabowska, Grzegorz Mazur, Malgorzata Wieczorek, Maria Ejma, Krzysztof Slotwinski, Slawomir Budrewicz

**Affiliations:** 1 Department of Neurology, Wroclaw Medical University, Borowska 213, 50-556 Wroclaw, Poland; 2 Department of Internal Medicine, Occupational Diseases, Hypertension and Clinical Oncology, Wroclaw Medical University, Borowska 213, 50-550 Wroclaw, Poland; 3 Department of Hygiene, Wroclaw Medical University, J. Mikulicza-Radeckiego 7, 50-345 Wroclaw, Poland; 4 Faculty of Earth Sciences and Environmental Management, University of Wroclaw, Plac Uniwersytecki 1, 50-137 Wroclaw, Poland

**Keywords:** Cadmium, Lead, Arsenic, Polyneuropathy, Small fibers

## Abstract

**Background:**

Chronic exposure to heavy metals affects various organs, among them the brain and peripheral nerves. Polyneuropathy is mainly length-dependent with predominantly sensory symptoms. There have been few studies on small fiber neuropathy due to heavy metal intoxication.

**Methods:**

We investigated 41 metal industry workers, mean age 51.3 ± 10.5 years, with at least 5 years’ professional exposure to heavy metals, and 36 age- and sex-matched healthy controls. We performed neurological examinations, and assessed blood levels of cadmium, lead, and zinc protoporphyrin, urine levels of arsenic, standard, sensory and motor electrophysiological tests in the ulnar and peroneal nerves, sympathetic skin responses from the palm and foot, and quantitative sensation testing from dermatomes C8 and S1.

**Discussion:**

The results of standard conduction tests of all nerves significantly differed between groups. The latency of sympathetic skin responses achieved from the foot was also statistically significantly prolonged in the study group. Significant differences were seen in both C8 and S1 regions for temperature and pain thresholds, and for vibratory threshold only in the S1 region, while the dispersions of low and high temperatures were important exclusively in the C8 region.

**Conclusions:**

We can conclude that co-exposure to many heavy metals results in explicit impairment of peripheral nerves. The lesion is more pronounced within small fibers and is predominantly connected with greater impairment of temperature-dependent pain thresholds. The evaluation of small fiber function should be considered in the early diagnosis of toxic polyneuropathy or in low-dose exposure to heavy metals.

## Introduction

Chronic exposure to heavy metals affects various organs, including the brain and peripheral nerves. Heavy metal intoxication is a well-known cause of peripheral neuropathy. The clinical picture of such toxic polyneuropathies usually matches a sensoro-motor pattern with paresthesias, and in some cases weakness of the distal muscles, e.g. length-dependent neuropathy with predominant sensory symptoms [1–4]. Acute onset is rare, but possible [5]. Arsenic polyneuropathy is mainly characterized by axonal damage to sensory nerves [6] and is found in above 95% of patients. Motor neuropathy with a mainly demyelinating pattern, as an exception among others, is classically attributed to chronic lead intoxication [7–10], but there are literature items indicating the possibility of mild sensory and autonomic fiber dysfunction [9, 11], as well as mixed damage to peripheral nerves [12]. Cadmium poisoning is mainly connected with behavioral changes [13, 14], and there are few studies on possible polyneuropathy [15]. These three heavy metals are among the most common toxic metals and co-exposure to them in high concentrations occurs mainly in the metal industry. Despite the protection of employees, prolonged occupational exposure to heavy metals may cause toxic symptoms [16–18].

Standard electrophysiological methods allow estimation of conduction parameters in peripheral nerves, and in sensory and motor fibers, but only in those of the largest diameter [1, 19–21]. In toxic polyneuropathies, sensory fibers seem to be more often damaged than motor ones, not so infrequently subclinically, or the impairment applies only to small fibers [22–25]. The additional assessment of small fiber function could be useful in the diagnostics of subclinical toxic polyneuropathy. Quantitative sensation testing (QST) allows evaluation of thermal and vibratory perception. Based on the determination of cold, warm and temperature-induced pain or vibratory thresholds, the function of small A-delta, and C fibers could be assessed together with larger fibers — A-beta. Additionally, the evaluation of autonomic fiber function is commonly used in the examination of small fiber polyneuropathy [1, 26, 27].

The aim of this study was the electrophysiological evaluation of peripheral nerves with regard to the function of small fibers in workers occupationally co-exposed to arsenic, cadmium, and lead. Attention was paid to the eventual correlations between toxicological and electrophysiological parameters in order to estimate the pattern of electrophysiological changes to peripheral nerves.

## Materials and methods

The Ethics Committee of Wroclaw Medical University in Poland gave its approval for the study. Informed consent for participation in the study was obtained from all volunteers.

We investigated 41 employees of the “Legnica” and “Głogów” Copper Smelters and Refineries (Poland), 40 men and 1 woman, mean age 51.3 ± 10.5 years. The average weight in the study group was 85.22 ± 10.72 kg, and body mass index (BMI) was 27.63 ± 2.97 kg/m^2^ All participants had documented professional exposure to heavy metals for at least 5 years, subject to the use of professional security methods. We excluded all participants with additional diseases and conditions influencing the peripheral nervous system: diabetes mellitus, hormonal, rheumatic, and malignant diseases, vitamin deficiency, addiction to alcohol and drugs, and current professional exposure to chemical substances other than heavy metals and physical burdens. The control group consisted of 36 healthy individuals not professionally or incidentally exposed to heavy metals, age- and sex-matched (35 men and 1 woman, 51.1 ± 12.7 years old), with similar body weight (80.50 ± 9.12 kg, *p* > 0.05) and BMI (26.68 ± 1.81 kg/m^2^, p > 0.05).

Standard employment and medical histories with information including lifestyle were collected from all participants. Clinical neurological examination, and biochemical tests were performed. In the toxicological analysis we estimated: blood levels of cadmium (Cd-B), and lead (Pb-B) and urine levels of arsenic (As-U). Zinc protoporphyrin (ZnPP) level was evaluated as a toxic effect marker of Pb. Values of Cd-B higher than – 5 μg/l, Pb-B – 500 μg/l, ZnPP – 70 μg/dl, and As-U – 35 μg/ g creatinine were considered the highest acceptable concentrations. Pb-B and Cd-B were estimated by use of atomic absorption spectroscopy in a graphite furnace with a Solaar M6 apparatus, Thermo Elemental, UK. For As-U estimation, atomic absorption spectroscopy using the hydride generation method with a VP100 Continuous Flow Vapor System was used. The analysis of ZnPP concentration in erythrocytes was evaluated using a fast fluorimetric method with a hematofluorimeter, ProtoFluor Helena (USA). All toxicological tests were carried out according to the standards of the Institute of Occupational Medicine in Łódź.

Electrophysiological studies were performed with a Viking Quest version 10.0 device connected with a Thermal Sensory Analyzer II 2001 (TSA II) (Medoc, Israel), Viking Select version 7.1.1c., and a Nicolet Biomedical device with the Multi-Mode Program (MMP Plus) software. All tests were performed at a room temperature of between 21 and 23 °C, skin temperature was equal to or above 32 °C.

In the nerve conduction study (NCS), we evaluated motor and sensory parameters in the ulnar and peroneal nerves (sural nerve for sensory conduction estimation in the lower limb) on the left side. We analyzed distal latency (in milliseconds – ms), amplitude (in millivolts – mV for motor potentials, in microvolts –μV for sensory potentials), and conduction velocities (in meters per second – m/s), using standard methods [19, 20]. The distances between electrodes and points of motor fiber stimulations at the wrist and ankle were standard: 5.5 cm and 8 cm, respectively. We analyzed motor conduction velocity along the nerves on the forearm (wrist – elbow) and on the calf (ankle – fibula). For sensory conduction evaluation, we used the antidromic method. The sensory responses were obtained from the fifth digit for the ulnar nerve, and from the lateral ankle region for the sural nerve. The distances between stimulating and recording electrodes varied from 11 to 13 cm for both nerves. The duration of the electrical stimulation was 0.2 ms.

Sympathetic skin responses (SSR) for electrical stimuli were assessed from the left hand and foot. Unexpected square electrical impulses of 10 to 30 mA were used. We stimulated the wrist region on the opposite side. We analyzed the shortest latency, and the amplitude of SSR, but for the statistical analysis used only the latency parameter [19, 27, 28].

QST allowed us to estimate the sensation and pain thresholds for cold and warm temperatures (cold sensation – CS, warm sensation – WS, cold pain – CP, heat pain – HP), and additionally the vibration threshold (vibratory sensation – VS). We also analyzed the temperature differences (dispersion of temperature); that is, the difference between feeling pain and temperature sensation (CS - CP, and HP - WS), which reflects dispersion of the temperature. We applied limit methods in the QST study. The thermode was attached to the skin on the region of the C8 dermatome (hypothenar region) for the ulnar nerve, and for the S1 dermatome (upper-lateral surface of the foot) for the sural nerve. The temperature changed by 1 °C/s for temperature threshold, and by 2 °C/s for pain threshold estimation. The adaptation temperature was constant and equal to 32 °C. Vibratory stimulation was performed using a special device module. The patients put their little finger (the ulnar nerve) and little toe (the sural nerve) on a vibrating button. The stimulation parameters were: rate - 100 Hz, amplitude range - 0 to 130 μm (μ), and amplitude change rate - 0.3 μm per second (μ/s). The stimulation for all thresholds was stopped when the patient pressed a button. The procedures were repeated 4 times for temperature, 3 times for pain, and 6 times for vibration threshold estimation. The thresholds were calculated as the average values, in °C and μ, respectively [26, 29, 30].

Statistical analysis was performed with *STATISTICA* 12.0 software (Statsoft Polska Sp. z o.o., Krakow, Poland). The statistical analysis included: the number of cases (N), and mean values (X) with standard deviations (SD) of the continuous parameters. For data with a normal distribution and homogeneity of variance the student t-test was used; for those with a normal distribution but without homogeneity of variance, the Cochran-Cox test was employed. The Mann-Whitney U was used for data without a normal distribution, and this concerned most of the calculated parameters. The normality of distribution was assessed using the Shapiro-Wilk test. The significant *p*-value was ≤0.05. Based on Guilford’s interpretation of the magnitude of significant correlations.

## Results

All volunteers met the inclusion / exclusion criteria. All underwent neurological examinations. Only 8 patients (19.5%) had the clinical symptoms of polyneuropathy, i.e. impaired sensation and / or diminished or absent deep reflexes in the distal localization (both were seen in 3 persons). In all of these, abnormalities were found only in lower limbs.

Basic biochemical analyses were within normal limits. The average level of As-U was 15.60 ± 15.58 μg/g creatinine, and in 4 persons the level was above 35 μg/g creatinine. Cd-B mean level was 1.19 ± 1.40 μg/l, Pb-B - 181.85 ± 139.88 μg/l, and for both metals only in 1 volunteer did the level exceed the higher acceptable concentration. This patient with a high Cd-B value (7.17 μg/l) also had a high level of As-U (88 μg/g creatinine). The average concentration of ZnPP was 18.35 ± 22.00 μg /dl, while in 2 persons it was higher than 70 μg/dl.

Standard motor and sensory conduction tests of the peripheral nerves differed statistically significantly in terms of nearly all parameters in the peroneal nerve, while in the ulnar nerve the differences were less obvious. All values are shown in Tables 1 and 2 for motor and sensory conductions, respectively.

**Table 1:** Motor conduction parameters in the ulnar and peroneal nerves in the study and control groups

nerve/parameter		study group *n* = 41		control group *n* = 36		*p-*value
		mean	SD	mean	SD	
Ulnar	latency (ms)	2.7	0.37	2.6	0.43	0.0854
	amplitude (mV)	9.4	2.09	9.1	2.28	0.3287
	conduction velocity (m/s)	56.3	4.75	58.9	5.75	0.0375
	F wave (ms)	28.2	1.60	27.2	1.56	0.0105
peroneal	latency (ms)	5.0	1.04	4.5	0.80	0.0011
	amplitude (mV)	5.4	1.77	8.3	2.88	0.0001
	conduction velocity (m/s)	45.7	3.20	47.6	4.31	0.0379
	F wave (ms)	50.1	3.11	49.1	1.96	0.0026

*ms* milliseconds, *mV* millivolts, *m/s* meters per second

**Table 2:** Sensory conduction parameters in the ulnar and sural nerves in the study and control groups

nerve/parameter		study group *n* = 41		control group *n* = 36		*p-*value
		mean	SD	mean	SD	
ulnar	latency (ms)	2.3	0.24	2.2	0.38	0.1007
	amplitude (μV)	20.6	6.88	25.3	11.51	0.0109
	conduction velocity (m/s)	51.2	6.12	57.0	7.16	0.0005
sural	latency (ms)	2.80	0.54	2.38	0.49	0.0022
	amplitude (μV)	9.8	3.92	14.6	6.13	0.0002
	conduction velocity (m/s)	51.7	10.00	50.3	6.57	0.0666

*ms* milliseconds, *μV* microvolts, *m/s* meters per second

The latency of SSR achieved from the hand was 1.50 ± 0.28 ms in the study group versus 1.46 ± 0.13 in the control group, *p* = 0.0465. The SSR latency achieved from the foot was 2.44 ± 0.51 ms and 2.13 ± 0.21 ms, *p* < 0.0000, respectively.

**Fig. 1:**
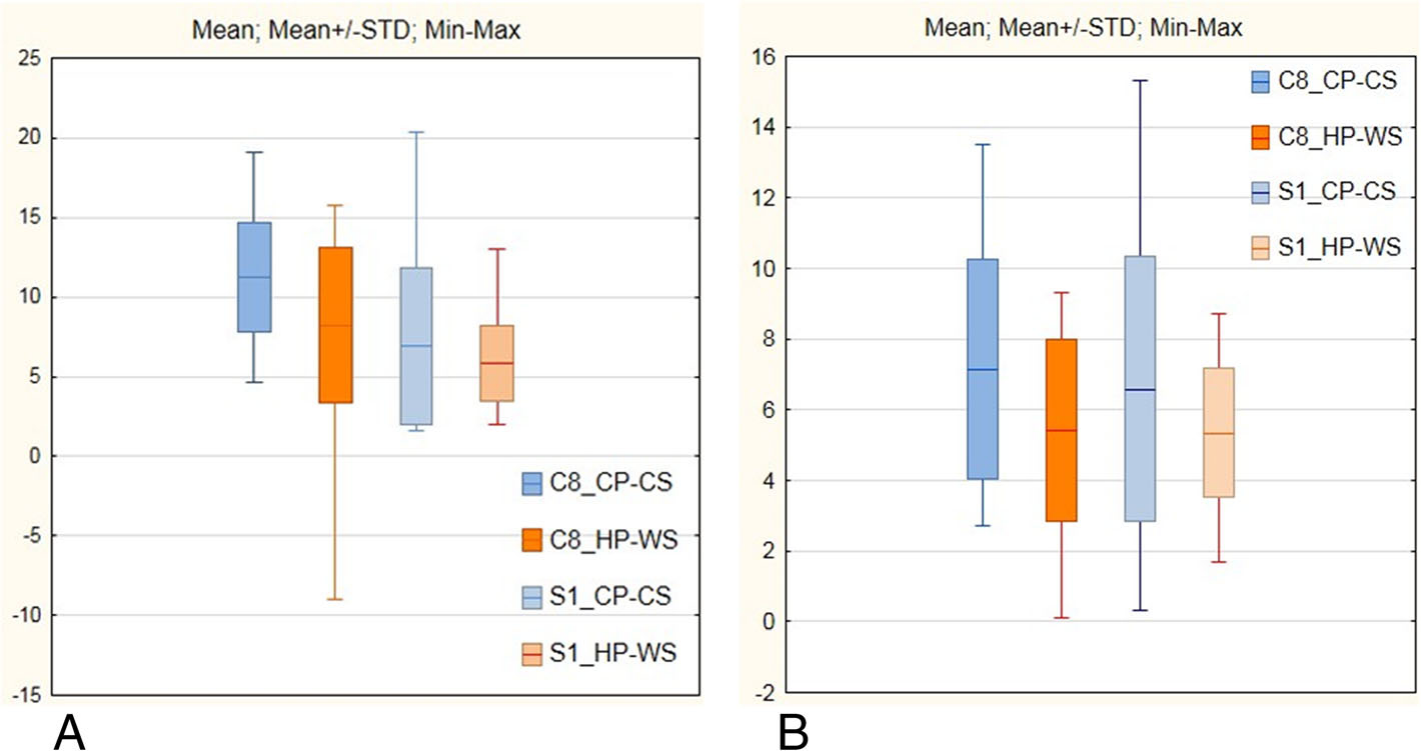
Dispersion (the temperature difference between feeling pain and temperature sensation) of low (cold) and high (warm) temperatures (in °C) in the C8 and S1 skin regions in metal industry workers (**a**) and controls (**b**). CS - cold sensation, WS - warm sensation, CP - cold pain, HP - heat pain

Analysis of sensation thresholds in the different modalities in the C8 and S1 skin regions revealed significant differences between study and control groups (Tables 3 and 4). Differences were seen in both C8 and S1 regions for temperature and pain thresholds, and for the vibratory threshold only in the S1 region. In the upper limb, the vibratory threshold was similar in both groups. We assessed the dispersions of low (cold) and high (warm) temperatures in the C8 and S1 skin regions. Statistically significant differences were seen only in the C8 region, but for both cold and warm sensations (Table 5, Fig. 1).

**Table 3:** Mean QST parameters in C8 in the study and control groups

C8 region/parameter	Study group *n =* 41		Control group *n =* 36		*p-*value
	mean	SD	mean	SD	
cold sensation (°C)	29.4	0.91	29.8	1.02	0.0076
warm sensation (°C)	34.7	0.93	34.2	0.87	0.0098
cold pain (°C)	17.3	6.44	23.2	3.41	0.0000
hot pain (°C)	43.2	4.17	39.6	2.98	0.0000
vibratory limits (μ)	2.1	1.57	1.8	0.74	0.9911

*°C* degrees Celsius, *μ* microns

**Table 4:** Mean QST parameters in S1 in the study and control groups

S1 region/parameter	Study group *n =* 41		Control group *n =* 36		*p-*value
	mean	SD	Mean	SD	
cold sensation (°C)	26.5	2.08	28.5	1.92	0.0001
warm sensation (°C)	38.8	3.20	36.5	2.01	0.0011
cold pain (°C)	18.9	7.24	21.9	5.01	0.0012
hot pain (°C)	44.6	3.01	41.9	1.87	0.0000
vibratory limits (μ)	7.1	6.44	2.8	1.16	0.0001

*°C* degrees Celsius, *μ* microns

**Table 5:** Dispersion (the temperature difference between feeling pain and temperature sensation) of low (cold) and high (warm) temperatures (in °C) in the C8 and S1 skin regions

Skin region/parameter	study group *n =* 41		control group *n =* 36		*p-*value
	mean	SD	Mean	SD	
C8 cold dispersion (°C)	11.25	3.47	7.14	3.10	< 0.0001
C8 warm dispersion (°C)	8.24	4.86	5.41	2.58	0.0015
S1 cold dispersion (°C)	6.95	4.91	6.59	3.76	0.7849
S1 warm dispersion (°C)	5.85	2.32	5.34	1.81	0.3153

*°C* degrees Celsius

The correlations between the analyzed parameters of standard neurography, SSR, TSA and levels of heavy metals were not generally significant. Only a few of these showed a weak relationship (maximal value 0.48). They did not indicate any significant regularities in relation to individual metals and did not allow us to estimate any pattern of polyneuropathy for the individual factors. When we compared sensation thresholds in the different modalities in the C8 and S1 skin regions in patients with Cd-B, Pb-B, Zn-PP and As-U levels above cut-off values (Table 6), we found that the patient with high Pb-B level had the highest thresholds of temperature and pain sensation; CP thresholds in both regions were completely undetectable. High Zn-PP values were not connected with significantly higher thresholds, which were around mean values in the study group. The results found in the patient with high levels of both Cd-B and As-U were higher than the average results in the study group, but not as high as those in the aforementioned patient with significantly elevated Pb-B level. The highest vibratory thresholds, mainly in the S1 region, were seen in those patients with abnormal As-U levels. The temperature dispersions (the temperature difference between feeling pain and temperature sensation) for low and high temperatures in those patients with Cd-B, Pb-B, Zn-PP or As-U levels above the cut-off values were generally smaller in the lower limbs than in the upper limbs, similar to the control group.

**Table 6:** Heavy metals levels and TSA results in the study group (mean values) and in 7 patients with high levels (above cut-off value) of Cd-B, Pb-B, Zn-PP or As-U

	Cd-B (μg/l)	PB-B (μg/l)	As-U (μg/g creat.)	Zn-PP (μg/dl)	C8							S1						
					CS (°C)	WS (°C)	CP (°C)	HP (°C)	VS (μ)	CS-CP	HP-WS	CS (°C)	WS (°C)	CP (°C)	HP (°C)	VS (μ)	CS-CP	HP-WS
study group	1.19	181.85	15.60	18.35	29.4	34.7	17.3	43.2	2.1	11.3	8.24	26.5	38.8	18.9	44.6	7.1	6.9	5.9
Patient 1	7.17	374	88	50	30.3	34.8	19.7	49.5	1.1	10.6	14.7	29.6	41.7	21	46.6	1.9	8.6	4.9
Patient 2	0.42	211	42.1	8	28.8	35.1	19.6	46.9	1.5	9.2	11.8	26.5	40.5	23.1	44.9	2.6	3.4	4.4
Patient 3	1.74	322	44.8	11	30.2	33.6	21.5	41.7	1.1	8.7	8.1	26.4	36.3	24.1	42.1	5.1	2.3	5.8
Patient 4	0.21	610	3.84	9	29.5	36.2	^a^	49.6	1.9	^b^	13.4	27.5	44.8	^a^	49.8	1.2	^b^	5.0
Patient 5	0.43	409	45.8	4	29.7	33.5	12.9	44.5	2.4	16.8	10.0	23.1	43.5	21.1	47.4	10.9	2.0	3.9
Patient 6	0.29	384	22.1	88	30.4	34.9	15.3	45.7	1.2	15.1	10.8	29.9	42.8	19.8	48.6	8.4	10.1	5.8
Patient 7	0.48	66.7	3.9	106	28.8	34.1	20.7	43.9	0.7	8.1	9.8	27.8	34.9	22.1	42.6	1.6	5.7	7.7

^a^undetectable

^b^impossible to determine

## Discussion

Exposure to heavy metals in high concentrations mainly occurs in the metal industry. Co-exposure to several heavy metals, not infrequently including other occupational factors, is highly likely in this industry. The effects on e.g. DNA, carcinogenesis, and faetal development as a result of co-exposure to toxins seem to exceed the sum of effects of single metals [16, 17, 31–33]. However, it is also possible that co-exposure to some metals (As and Pb, Pb and Cd) has antagonistic effects [6, 34]. The influence of co-exposure to heavy metals on the peripheral nervous system is equivocal [18]. In our study group, less than 20% of volunteers had clinical symptoms of polyneuropathy. This allowed us to recognize a length-dependent pattern of polyneuropathy, mostly with dominant sensory disturbances. Standard NCS testing of motor and sensory fibers confirmed peripheral nerve damage. Changes were found in upper and lower limbs in respect of sensory, as well as motor fibers. We found the electrophysiological features of both axonal lesion and demyelination. Even though we did not consider the sensory latency values, motor latency and motor and sensory conduction velocities resulting from demyelination differed significantly from the control group, as did amplitude values (axonal lesion). *P*-values for most sensory parameters were much lower (*p* > 0.001) than for motor ones, and in the peroneal nerve compared to the ulnar nerve; typically, more pronounced changes were seen within sensory fibers, and in the lower limbs.

In their longitudinal study using NCS, Ishii et al. [35] confirmed sensory impairment in upper and lower limbs with only slight motor dysfunction after arsenic intoxication. Mixed, axonal and demyelinating peripheral neuropathy could develop as a subacute damage [22, 36–40] and even be found after drinking water contaminated with a very low dose of arsenic [41]. On the other hand, in some studies only subclinical forms of arsenic polyneuropathy have been noted after occupational, long-lasting exposure [42], and this usually takes the form of axonal sensory neuropathy [37, 43].

Cadmium is a known, potent toxin for cortical neurons, even in low doses [44]. Cadmium intoxication can lead to olfactory, neurobehavioral, and memory problems, and may underlie neurodegeneration [4, 13, 45–47]. Little is known about the influence of cadmium on the peripheral nervous system [15, 48]. The adverse effects of cadmium on the auditory system have been described in some studies [18]. Viaene et al. [15] suggested the promoting role of cadmium in the development of polyneuropathy at an older age.

Lead neuropathy has been described by many authors, with the classical predomination of muscle weakness in forearm extensors with very slight sensory involvement [7, 9, 11, 40, 43, 49]. Distal, symmetric, sensory and motor polyneuropathy occur less commonly in lead intoxication [8, 9, 11]. Classical lead neuropathy is usually subacute and develops after relatively short exposure to high lead concentrations, while chronic exposure to inorganic lead leads to mild sensory and autonomic symptoms [7, 8]. Based on the determination of DNA single stand break (DNA-SSB) induction and repair capacity for 8-oxoguanine in mononuclear blond cells, Hengstler et al. [31] proved the interactive effects of co-exposure to cadmium, cobalt and lead, and their genotoxic effects even in non-critical concentrations. In another study, Xue et al. [16] confirmed that chronic exposure to metal mixtures may increase these toxic effects, but on the other hand co-exposure to specific metals, e.g. As and Pb, may have antagonistic effects leading to a reduction of toxicity.

Little is known about the effect of co-exposure to heavy metals on the peripheral nervous system. For our study, we included workers with prolonged professional exposure to different heavy metals – Pb, Cd, As. Our neurophysiological findings confirmed peripheral nerve damage in all of them. Clinically, polyneuropathy was seen in 20%, while standard neurophysiological tests in the majority of the results achieved from lower limbs were incorrect. We were able to conclude that co-exposure to combinations of the above heavy metals produced more pronounced and more easily identifiable neuropathy. The additive influence of mixed heavy metals on peripheral nerves is possible, even though one of our patients with both Cd-B and As-U levels above cut-off values had elevated, but not the highest, temperature and pain thresholds. Additionally, during our evaluation of the function of small fibers of peripheral nerves, we more clearly noted abnormalities in our patients. Nearly all parameters significantly differed between groups, and the electrophysiological signs of polyneuropathy were also clearly positive in the upper limbs. All our investigations confirmed the lesion of all fiber types, large and small, in the lower limbs. Greater differences in the dispersion of low and high temperatures, which resulted from the higher thresholds of pain sensation, were found only in the C8 region. This was also confirmed in 7 patients with high Cd-B, Pb-B, Zn-PP and As-U levels, which were above cut-off values. This allowed us to state that small fibers (A-delta and C) are damaged earlier than large fibers. Estimation of their function should be considered in the early diagnosis of toxic polyneuropathy or in low-dose exposure to heavy metals. Our conclusions remain consistent with those of many previous studies. A chronic length-dependent sensorimotor polyneuropathy, in many cases presenting as sensory-predominant painful neuropathy, is seen in the toxicity of heavy metals [1, 2, 5, 6, 10, 15, 21, 40, 50]. However, sensory disturbances in patients with chronic arsenic exposure seem to be related not only to peripheral neuropathy but also to impairment of the central nervous system [51]. Gobba et al. [23, 24] proved that exposure to industrial metals could affect different sensory functions: hearing, taste, etc. Marcetti [52] underlined dysfunction in smell with cadmium and nickel toxicity. Chronic exposure to arsenic in drinking water, even in low concentrations, can produce objective neuropathies in both large and small fibers [41]. Considering our two patients with high As-U values we can state more pronounced changes in vibratory thresholds, thus in large diameter sensory fibers. Predominantly, sensory polyneuropathy together with autonomic symptoms is observed in 48% of patients exposed to arsenic [53]. In chronic low-level lead intoxication, an impact on unmyelinated small fibers has also been proved in individual studies [54].

The limitations of our study included the small number of participants, and the lack of control groups consisting of patients exposed to single heavy metals separately. We were unable to find any significant correlation between parameters for large and small fiber dysfunction and the concentrations of the individual heavy metals. Therefore, we could not find or match any specific neurophysiological pattern to the respective heavy metals – Cd, As, and Pb.

## Conclusions

We can conclude that co-exposure to many heavy metals results in explicit impairment of peripheral nerves, which is more pronounced within small fibers. We can state that the most interesting finding was the dispersion of low and high temperatures, which was predominantly connected with greater impairment of temperature-dependent pain thresholds. We confirmed that QST is a sensitive test, and allows the demonstration, with a high degree of sensitivity, of even slight impairment to small fibers in toxic neuropathy. The evaluation of small fiber function should be considered in the early diagnosis of toxic polyneuropathy or in low-dose exposure to heavy metals. Our results did not allow us to assign our neurophysiological findings to the influence of a particular heavy metal or its level. Based on the literature review, we can state that few studies have been conducted into small fiber neuropathy due to heavy metal intoxication, so our study seems to be an important, additional element in the field of the medicine of industrial toxicology.

## Data Availability

The datasets used and analyzed during the current study are available from the corresponding author on reasonable request.

## Abbreviations

μ/s: Microns per second
μV: Microvolts
As: Arsenic
As-U: Urine levels of arsenic
BMI: Body mass index
Cd: Cadmium
Cd-B: Blood levels of cadmium
CP: Cold pain
CS: Cold sensation
DNA-SSB: DNA single stand break
HP: Heat pain
m/s: Meters per second
MMP Plus: Multi-Mode Program
ms: Milliseconds
mV: Millivolts
NCS: Nerve conduction study
Pb: Lead
Pb-B: Blood levels of lead
QST: Quantitative sensation testing
TSA II: Thermal Sensory Analyzer II
VS: Vibratory sensation
WS: Warm sensation
ZnPP: Blood levels of zinc protoporphyrin
